# Institutional delivery service utilization and associated factors among women of reproductive age in the mobile pastoral community of the Liban District in Guji Zone, Oromia, Southern Ethiopia: a cross sectional study

**DOI:** 10.1186/s12884-017-1325-5

**Published:** 2017-05-15

**Authors:** Wako Golicha Wako, Dejene Hailu Kassa

**Affiliations:** 1 Wolkite University College of Medicine and Health Sciences, Welkite, Ethiopia; 2 Hawassa University College of Medicine and Health Sciences, Awasa, Ethiopia

**Keywords:** Institutional delivery, Mobile pastoralists, Indirect health care cost

## Abstract

**Background:**

Globally, an estimated 289,000 maternal deaths occurred in 2013. Majority of these deaths occurred in sub-Saharan Africa and Southern Asia. Mobility of pastoralists is a well-recognized survival strategy in arid and semi-arid land of sub-Saharan Africa. However governments often encourage settlement as a solution to the difficulty of providing health services for mobile pastoralists. This study aimed to assess utilization of institutional delivery and associated factors among women of reproductive age in the mobile pastoral community of the Liban District in Guji zone, Oromia, Ethiopia.

**Methods:**

A Community based cross-sectional survey was conducted among the mobile pastoralist community of the Liban District. Seven hundred ninety-one (791) randomly selected women, who had birth within the last 2 years preceding the survey, were interviewed using a pretested structured questionnaire. Data were entered into Epi-Info version 3.5.4 and analyzed by Statistical Package for Social Science (SPSS) version 16. Bivariate and multivariate analyses were done.

**Results:**

Out of 791 women who gave birth within the last 2 years preceding the survey, only 110 (13.9%) gave birth in health institutions. Majority (74.1%) of the women gave birth at their home. Ninety-one women (11.5%) gave birth at traditional birth attendant’s home; assisted by traditional birth attendants. Multiple logistic regression shows that women who had readily available cash at the onset of labor (aOR 2.79, 95% CI: 1.29–6.25), delivered the birth preceding the most recent birth in a health institution (aOR 6.8, 95% CI: 3.44–13.45) and had birth related complications during the birth preceding the most recent birth (aOR 1.90, 95% CI: 1.08–3.36) were more likely to deliver at health institutions.

**Conclusion:**

Majority of the pastoral women seek institutional delivery, only when labor related complications are perceived. Mechanisms of alleviating indirect health care costs affecting institutional delivery need to be addressed in future studies.

**Electronic supplementary material:**

The online version of this article (doi:10.1186/s12884-017-1325-5) contains supplementary material, which is available to authorized users.

## Background

At the beginning of this century, 189 member states of United Nations endorsed a millennium declaration and committed themselves to eight Millennium Development Goals (MDGs) to improve the living standards of citizens worldwide [1]. Two of these goals were to reduce child mortality (MDG 4) and maternal mortality (MDG 5) [1]. By the end of 2015 some of these MDGs like poverty reduction and primary education for all were achieved. However achieving MDG 4 and MDG 5 were failed especially in sub-Saharan Africa [2].

Globally, it was estimated that 289,000 maternal deaths occurred in 2013 [3]. This indicates a 45% reduction from the baseline of 1990 [3]. According to the 2013 World Health Organization (WHO) report, maternal deaths are highest in sub-Saharan Africa and Southern Asia. Sixty two (62%) percent of global maternal deaths took place in sub-Saharan Africa alone, followed by southern Asia (24%) [3]. Ethiopia is among countries with highest maternal mortality ratio (MMR) in the world with an estimated MMR of 676/100,000 live births [4].

In the past century, pastoral zones have been neglected by governments in terms of social services provision including health care. Geographical dispersion and mobility of pastoralists would create significant barriers to health service delivery, including institutional delivery. Governments often encourage settlement as a solution to the difficulty of providing health services for mobile pastoralists [5]. Mobility of pastoralists, however, is a well-recognized survival strategy in arid and semi-arid lands of sub-Saharan Africa. Moreover mobility is very critical for the livestock survival and, the livelihoods and survival of the pastoralists [6].

Many studies explored health institutional delivery utilization and its predictors in most socio-economic settings of Ethiopia. Few efforts, however, have been made to study this in a pastoral community. Therefore, this study aimed to assess the utilization of institutional delivery and associated factors among women of reproductive age in the mobile pastoral community of the Liban District in Guji Zone, Oromia.

## Methods

### Study setting

The study was conducted in the Liban District of Guji Zone, State of Oromia, Southern Ethiopia. The district is located in the lowlands of southern Ethiopia approximately 925 meters above sea level. The district’s administrative town, Nagelle Borana is located 595 km south of Addis Ababa, Ethiopia’s capital. The district is predominantly inhabited by the Borana agro-pastoral and pastoral community. Among the inhabitants are mobile pastoralists whose lifestyle is characterized by seasonal mobility for wise management of scarce water and pasture for a livestock.

### Study design and population

A community based cross-sectional survey was conducted among women of reproductive age who had given birth within the last 2 years preceding the survey. The study was conducted in the area of the district considered to host mobile pastoralists.

### Sample size and sampling procedures

The report of 2014 Ethiopian mini Demographic and Health Survey (EDHS) was used to estimate sample size for the first objective. According to the report 10.4% of rural women delivered in health institutions [7]. Considering 95% confidence level and 3% absolute precision; the minimum required sample size was calculated as following:- n = (z_1-_α/2) ^2^*p (1-p)/*d* ^2^ ; were: z_1-_α/2 = z-score corresponding to 95% confidence interval, which is 1.96; p- is anticipated sample proportion, which is 10.4% and d- is an absolute precision, which is 3%. Accordingly the minimum required sample size was 398. Sample size for the second objective was calculated by Epi-info7 statCalc software. Three independent variables were considered for the sample sizes calculation. The sample sizes were calculated with common assumptions of: Power of 80%; two sided confidence level of 95% and ratio of control to case of 1. Finally the largest sample size among four sample sizes was used for the study. Therefore sample size for the first objective (i.e. n = 398) was used, because it is the largest one. Because participants’ selection was not done by direct simple random sampling method, the design effect of 2 was considered. Additionally, 10% non-response rate was considered. Final sample size required was: 398 * 2 + 0.1(398*2) ≈ 876.

### Sampling procedures

The area of the district which hosts majority of the mobile pastoralists is found to one side of the district. This side of the district was considered as one geographical area. The geographical area is formed by four different kebeles. The Sample size was allocated to the kebeles proportional to the approximate number of households. Each kebele was further subdivided into smaller administrative units called “Gare”. Finally, the sample size taken from each “Gare” was allocated proportional to the number of households in the “Gare”. In each “Gare” eligible women were enumerated and participants were selected by systematic random sampling. The whole process of approximating households, allocating sample sizes to each kebele and “Gare”, selecting the participants and data collection was accomplished within three weeks to reduce effects of mobility on the study.

### Data collection

Data was collected by a pretested structured questionnaire. An English language questionnaire was developed by authors after reviewing relevant literatures. Finalized version of the questionnaire was translated into local language (i.e. Afan Oromo). The Afan Oromo questionnaire was pretested in nearby pastoral kebele not participating into the study. The Afan Oromo questionnaire was used to collect data. Informed verbal consent to participate was obtained from participants. Verbal consent was preferred over written consent, because majority of participants were non-literate women.

### Data analysis

The data were entered into Epi-Info version 3.5.4, cleaned and analyzed with SPSS version 16. Frequency distributions were run and the prevalence of institutional delivery utilization was estimated. Bivariate analysis was done for all independent variables. *P* < 0.1 on bivariate analysis was used as a cutoff point to select the independent variables for multivariate analysis. Adjusted odds ratio with 95% confidence interval (CI) and *P* < 0.05 was used to declare statistical significance.

## Results

### Socio-demographic characteristics of the respondents

Out of the total 876 reproductive age women planned for the study, 791 women were successfully interviewed and included into final analysis (response rate 90.3%). Two questionnaires were excluded from analysis because of major incompleteness of the collected data. Eighty three (83) women were not found after frequent home visits. The mean (±SD) age of the respondents was 28.1 (±7) years. Majority of women were non-literate of any language (92.7%) and the rest (7.3%) were literate. Waqefata was dominant religion (59.3%) followed by Islam (32.5%) and Protestantism (8.2%). Majority of women were housewife (Table 1).

**Table 1:** Socio-demographic characteristics of the respondents, Liban District, Oromia; Southern Ethiopia; June 2015

Characteristics of respondents	Frequency	percentage
Age of women in completed year		
< =19	89	11.3
20–34	521	65.9
> =35	181	22.9
Total	791	100.0
Husbands’ educational level		
No education(grade 0)	575	83.7
Primary education(grade 1–8)	94	13.7
Secondary education and above ( grade 9 and above )	18	2.6
Total	687	100.0
Women’s occupation		
House wife	775	98.0
Others	16	2.0
Total	791	100.0
Family size		
< =4	163	20.6
5–6	176	22.3
> =7	452	57.1
Total	791	100.0
Months elapsed since last mobility by households		
< =5 months	59	7.5
6–59 months	408	51.6
> =60 months	324	41.0
Total	791	100.0

Approximately one third of the women owned ten or more “Hawicha” of cattle and 12% of the women owned ten or more “Hawicha” of camel. Table 2 shows details of household livestock ownership status.

**Table 2:** Livestock ownership by households, Liban District, Oromia, southern Ethiopia, June 2015

Types of livestock	Frequency	Percentage
Cattle owned in “Hawicha^a^”		
< =4	243	30.7
5–9	300	37.9
> =10	248	31.4
Total	791	100.0
Camel owned in “Hawicha^a^”		
< =4	459	58.0
5–9	237	30.0
> =10	95	12.0
Total	791	100.0
Goat and Sheep owned in “ Hawicha^a^”		
< =10	386	48.8
11–20	309	39.1
> =21	96	12.1
Total	791	100.0

^a^ “Hawicha”- is the local livestock counting mechanism, in which only animals which had at least one birth are counted

### Past obstetric history

Majority of the respondents, 724 (91.5%) were women who had more than one birth. Two hundred and twenty eight (31.5%) of women who had had more than one birth, reported complications during the birth preceding the most recent one. The most frequently reported complication was long duration of labor (see Table 3).

**Table 3:** Past obstetric history of respondents, Liban District, Oromia; southern Ethiopia, June 2015

Past obstetric history	Frequency	Percentage
Number of previous birth		
1	67	8.5
2–4	273	34.5
> =5	451	57.0
Total	791	100.0
Place of the birth preceding the most recent one		
Home	516	71.3
Traditional birth attendant’s home	92	12.7
Health post	8	1.1
Health center	88	12.2
Hospital	19	2.6
Others	1	0.1
Total	724	100.0
Presence of complication during the birth preceding the most recent one		
No	496	68.5
Yes	228	31.5
Total	724	100.0
Type of complication encountered during birth preceding the most recent one^a^		
Prolonged labor	126	50.4
Excessive vaginal bleeding	30	12.0
Still birth	47	18.8
convulsion	43	17.2
Others	4	1.6
Total	250	100.0

^a^: multiple responses are possible

### Knowledge and attitude of respondents towards maternal health services

Majority of women knew that government health institutions provide delivery service (92.7%). They knew about availability of free obstetric ambulance at Woreda health office (93.2%) and delivery services are provided free of charge at the government health institutions (90.1%).

### Institutional delivery utilization

Out of 791 women who gave birth within the last 2 years preceding the survey, only 110 (13.9%) gave birth in health institutions and the remaining 681 (86.1%) gave birth elsewhere. Among women who gave birth at the health institutions, only 30 (27.3%) had planned to give birth at a health institution. The remaining 80 (72.7%) gave birth at the health institutions because of labor related complications. Ninety-one (11.5%) of women gave birth at traditional birth attendants’ home and assisted by traditional birth attendants. Two hundred eighty six (36.2%) births were attended by traditional birth attendants, irrespective of place of delivery. Deliveries attended by skilled personnel were 102 (12.9%). One hundred seventeen (22.4%) women reported complications during labor and/or delivery of the most recent birth. The most frequently reported complication was prolonged duration of labor (Table 4).

**Table 4:** Characteristics of the most recent birth, Liban District, Oromia, Southern Ethiopia, June 2015

Characteristics of most recent birth	Frequency	Percentage
Place of the most recent birth		
Home	586	74.1
Traditional birth attendant’s home	91	11.5
Health post	10	1.3
Health center	75	9.5
Hospital	25	3.2
Others	4	0.5
Total	791	100.0
Who attended the most recent birth		
Relative	297	37.5
Traditional birth attendant	286	36.2
Health extension worker	10	1.3
Midwife/doctor/nurse/health officer	102	12.9
Nobody	96	12.1
Total	791	100.0
Type of complications faced during the most recent birth^a^		
Prolonged labor	105	53.6
Excessive vaginal bleeding	29	14.8
Still birth	23	11.7
Convulsion	32	16.3
Others	7	3.6
Total	196	100.0
Reasons for not delivering the most recent birth at a health institution^a^		
Facility too far/no transportation	355	43.7
Not customary	315	38.7
Do not know the existence of service	41	5.0
Do not trust health worker	41	5.0
Not necessary	17	2.1
Facility not open	14	1.7
No female provider	3	0.4
Others	27	3.3
Total	813	100.0

^a^: multiple responses are possible

When labor of the most recent birth started, 415 (52.5%) women had readily available cash. The remaining 335 (42.4%) women did not have readily available cash.

### Factors associated with institutional delivery

Odds ratios from multiple logistic regression show that women who had readily available cash when labor started (aOR 2.79, 95% CI:1.29–6.25), delivered the child preceding the most recent one in a health institution (aOR 6.8, 95% CI:3.44–13.45)and faced birth related complications during the birth preceding the most recent one (aOR 1.90, 95% CI:1.08–3.36) were more likely to deliver at a health institution. Utilization of institutional delivery among non-literate women was not different from literate women (aOR 0.92, 95% CI: 0.27-3.11). Institutional delivery utilization by women not in marital union was not different from those married (aOR 0.67, 95% CI: 0.24–1.57). Extent of household mobility, distance to road and health institution and perceived availability of transportation had no association with institutional delivery.

Radio listening frequency did not significantly affect the place of delivery. The utilization of institutional delivery among women who do not listen to radio at all was not significantly different from those who listen to radio sometimes (aOR 1.33, 95% CI: 0.67–2.61) and most of the time (aOR 1.02, 95% CI: 0.36–2.93) (Table 5).

**Table 5:** Socio-demographic factors associated with institutional delivery, results from logistic regressions, Liban District, Oromia, Southern Ethiopia, June 2015

Factors	Institutional delivery		Crude odds ratio	Adjusted odds ratio (95% CI)
	No (%)	Yes (%)		
Women age				
< =19	81 (91.0)	8 (9.0)	1.00	NA
20–34	452 (86.8)	69 (13.2)	1.56	NA
> =35	148 (81.8)	33 (18.2)	2.26	NA
Total	681	110		
Religion				
Waqefata	404 (86.1)	65 (13.9)	1.00	NA
Islam	219 (85.2)	38 (14.8)	1.08	NA
Protestant	58 (89.2)	7 (10.8)	0.75	NA
Total	681	110		
Women educational status				
Non-literate	631	102	1.00	NA
Literate	50	8	0.99	NA
Total	681	110		
Women marital status				
Not in marital union	81	23	1.00	NA
Married	600	87	0.51	NA
Total	681	110		
Months elapsed since last mobility by household				
< =5 months	53	6	1.00	NA
6–59 months	344	64	1.64	NA
> =60 months	284	40	1.24	NA
Total	681	110		
Time taken to reach nearest functional transport road				
< = one hour	455	96	1.00	1.00
> one hour	226	14	0.29	0.45 (0.16,1.26)
Total	681	110		
Time taken to reach nearest health facility				
< = 2 hours	169	12	1.00	1.00
3–5 hours	454	79	2.45	1.23 (0.47,3.22)
6 hours	68	19	4.61	2.26 (0.68,7.56)
Total	681	110		
Perceived availability of transportation when labor started				
No	386	54	1.00	1.00
Yes	295	56	1.38	0.58 (0.32,1.05)
Total	681	110		
Radio listening frequency				
Not at all	389	49	1.00	1.00
Sometimes	227	51	1.78	1.33 (0.67,2.61)
Most of time	65	10	1.22	1.02 (0.36,2.93)
Total	681	110		
Cattle owned in “Hawicha”				
< =4	217 (89.3)	26 (10.7)	1.00	1.00
5–9	246 (82)	54 (18)	1.83	1.23 (0.60, 2.53)
> =10	218 (87.9)	30 (12.1)	1.15	1.24 (0.53, 2.88)
Total	681	110		
Camel owned in “Hawicha”				
< =4	407 (88.7)	52 (11.3)	1.00	1.00
5–9	189 (79.7)	48 (20.3)	1.99	1.53 (0.80, 2.91)
> =10	85 (89.5)	10 (10.5)	0.92	1.38 (0.49, 3.89)
Total	681	110		
Goat and sheep owned in “Hawicha”				
< =10	340 (88.1)	46 (11.9)	1.00	NA
11–20	259 (83.7)	50 (16.3)	1.43	NA
> =21	82 (85.4)	14 (14.6)	1.26	NA
Total	681	110		
Readily available cash when labor started				
No	316 (94.3)	19 (5.7)	1.00	1.00
Yes	332 (80.0)	83 (20.0)	4.16	2.79 (1.25,6.25)*
Others^a^	33 (80.5)	8 (19.5)	4.03	5.57 (1.53,20.32)*
Total	681	110		
Husband’s education				
No education	502 (87.3)	73 (12.7)	1.00	NA
Primary education and above	98 (87.5)	14 (12.5)	0.98	NA
Total	600	87		
Family size				
< =4	146 (89.6)	17 (10.4)	1.00	NA
5–6	153 (86.9)	23 (13.1)	1.29	NA
> =7	382 (84.5)	70 (15.5)	1.58	NA
Total	681	110		
Decision maker for institutional delivery				
Husband and wife jointly	581 (86.6)	90 (13.4)	1.00	1.00
Mainly wife	57 (75.0)	19 (25.0)	0.46	(0.47, 12.66)
Total	638	109		

*NA* not applicable

*: the test was significant at *p*- < 0.05

^a^: either “I do not know” or “I do not remember”

Most obstetric characteristics of women had no association with institutional delivery, except the place of the birth preceding the most recent one and presence of complications during the birth preceding the most recent one (Table 6).

**Table 6:** Obstetric and related factors associated with institutional delivery, results from logistic regressions, Liban District, Oromia, Southern Ethiopia, June 2015

Factors	Institutional delivery		Crude odds ratio	Adjusted odds ratio (95% CI)
	No (%)	Yes (%)		
Parity				
< 4	210 (89.0)	26 (11.0)	1.00	NA
> =4	471 (84.9)	84 (15.1)	1.44	NA
Total	681	110		
ANC during pregnancy of the most recent birth				
No	237 (87.4)	34 (12.6)	1.00	NA
Yes	444 (85.4)	76 (14.6)	1.19	NA
Total	681	110		
Place of birth preceding the most recent birth				
Not health institution	561 (92.1)	48 (7.9)	1.00	1.00
Health institution	59 (51.3)	56 (48.7)	11.09	6.80 (3.44, 13.50)*
Total	620	104		
Presence of complication during birth preceding the most recent birth				
No	461 (92.9)	35 (7.1)	1.00	1.00
Yes	159 (69.7)	69 (30.3)	5.72	1.90 (1.08, 3.36)*
Total	620	104		
Willingness to deliver at facility where male provides service				
No	157 (94.6)	9 (5.4)	1.00	1.00
Yes , if no option	171 (85.5)	29 (14.5)	2.96	1.44 (0.45, 4.66)
Yes, no problem	352 (83.0)	72 (17.0)	3.57	2.25 (0.75, 6.72)
Total	680	110		
Attitudes towards institutional delivery				
Unfavorable	362 (89.2)	44 (10.8)	1.00	NA
Favorable	319 (82.9)	66 (17.1)	1.72	NA
Total	681	110		

*NA* not applicable

*: the test was significant at *p* <0.05

## Discussion

The prevalence of institutional delivery in this study was 13.9% (95% CI: 11.5–16.3%). Women who had readily available cash when labor onset, delivered the birth preceding the most recent one in a health institution and faced birth related complications during the birth preceding the most recent one were more likely to have institutional delivery.

Prevalence of institutional delivery in this study is similar with a study in Banja District, Amhara regional state of Ethiopia. In Banja District, 15.7% of women delivered in health institutions [8]. However, the prevalence in this study is much lower than the prevalence in Goba Woreda of Oromia regional state among urban and rural women where 47% (95% CI: 42.9–51.1%) delivered in health institutions [9]. In Ethiopia, evidences show that prevalence of institutional delivery among rural women is lower than among urban women [4, 7]. The current study shows lower prevalence of institutional delivery compared to the study in Goba Woreda, probably because of inclusion of urban women in the later. In addition to rural–urban difference, the difference in institutional delivery may be due to difference in socio-economic settings. Pastoral communities have limited access to social service infrastructures, including health care [5]. The limited access to health care might influence utilization of institutional delivery. However, the prevalence in the current study is a bit higher than the national rural average of institutional delivery in Ethiopia [7]. Mini Ethiopian Demographic and Health survey of 2014(EDHS 2014), reported that 10.4% (95% CI: 9.6–11.2%) of rural women gave their most recent birth in a health institution [7].

Women who had readily available cash at the time of labor onset were almost three times more likely to deliver at a health institution compared to those who did not have cash. Though delivery services are free of charge at government health institutions; indirect health care costs may be a barrier. Ninety percent of women who gave birth at the health institutions delivered at health centers or hospitals. Almost all of these health institutions are found at more or less urban areas, whether it is a small rural or urban town. Moving to such health institutions may demands rural women and/or accompanying persons to have at least some money to cover essential expenses like food. In pastoral communities, however, money consists of livestock, which might be difficult to sell any time unlike other assets. First, market centers where livestock are to be sold might be located far away at urban areas, requiring a long distance drive with their livestock to the market centers. Second, despite the distant market centers, market days are limited within a given week. In the study area, for instance there are only 2 market days per week. Third, because livestock are highly valued and major income resources, its sell may require discussion among family members. All these three above conditions make livestock an unreliable source of money during emergencies of a woman in labor, unless livestock is sold prior to onset of labor. This may be further evidenced by the fact that livestock possessions did not affect institutional delivery. This study did not assess birth preparedness and complications readiness, which includes capital preparation. A study in Goba Woreda showed that women who were birth prepared and had complication readiness were more likely to deliver at health institutions.

Provided that delivery services are given free of charge for all women, the current findings support the importance of indirect health care costs on institutional delivery. Work done in five low income countries showed that even in the presence of fee waiver and exemption systems women continue to pay for maternal health services, a large proportion of which is informal payments [10]. The same work demonstrated that fee waiver and exemption mechanisms will not alleviate the burden of out of pocket costs, because more than 80% of out of pocket costs for maternal health services are informal costs. Poor clients do not benefit from government fee subsidies because of poor awareness of fee waiver and exemption mechanisms [10]. In the current study, majority (84.2%) of the women had good knowledge of delivery service including free ambulance service and free delivery services at government health institutions. However, institutional delivery was not significantly different between women who had unfavorable and favorable attitudes towards institutional delivery.

The women who delivered the birth preceding the most recent birth at health institutions were almost seven times more likely to deliver at health institutions than those who delivered such birth elsewhere. The finding is in line with a study in Banja District of Amhara regional state, Ethiopia [8]. This may due to the fact that women who delivered at health institutions may better appreciate the advantages of institutional delivery, and this may encourage seeking health institutional delivery for subsequent deliveries.

The women who had complications during the birth preceding the most recent birth were more likely to deliver in health institutions. This finding is similar to a study in northern Ethiopia, which showed that women who had a history of obstetric complications tend to deliver at health institutions [11]. Also in current study, the majority of the women who delivered at the health institutions did so because of the complications they faced during labor. Among women who gave the most recent birth at the health institutions, only about 27.3% of them had planned to give birth at a health institution. The rest delivered at the health institutions because of perceived complications during labor.

Socio-demographic characteristics of women like age, literacy, religion and family size did not seem to affect the place of delivery. Place of delivery was not associated with current maternal age, consistent with a study in northern Ethiopia [11]. But it is inconsistent with many others which reported current maternal age as the factor affecting place of delivery [4, 7, 8]. Contrary to many studies, the current study reported that women’s literacy status did not an have association with place of delivery [7–9, 11, 12]. Consistent with many other studies, the current study reported that decision maker [8, 9, 11], husbands’ education [11] and habit of listening radio [8, 9, 11]^,^ did not significantly influence the choice of place of childbirth. Even though pastoral mobility was claimed as one cause of the difficulty in providing health care to pastoralists, the current study did not show a significant association between extent of household mobility and the place of childbirth. Similarly, distance to the nearest health institution and functional transport road were not associated with the place of childbirth. Similar findings were reported in a study from Goba Woreda [9]. However, 51.1% of the women who delivered outside health institutions mentioned lack of transportation and/or distance to health institution as one of the reasons for not delivering in a health institution.

Unlike many studies [7, 9, 12], antenatal care utilization was not associated with place of delivery in the current study. Also marital status and parity were not associated with the place of birth. These findings are consistent with studies done in Gondar and Goba, Ethiopia [9, 11]. In this study, the area of the district that considered hosting the mobile pastoralist and housing characteristics were criteria to distinguish mobile from settled pastoralists. But these criteria are not perfect in distinguishing the two from each other. So, chance of including some settled pastoral women in the study is possible. Therefore, this should be taken into account while interpreting the study.

## Conclusion

Majority of the pastoral women seek institutional delivery, only when labor related complications are perceived. Women should be educated to seek institutional delivery for all births. Mechanisms of alleviating indirect health care costs affecting institutional delivery need to be addressed in future studies.

## Additional file

Additional file 1: Questionnaire. Title of data: English language questionnaire for study entitled “Institutional delivery service utilization and associated factors among women of reproductive age in the mobile pastoral community of the Liban District in Guji Zone, Oromia, southern Ethiopia: A cross sectional study”. Description of data: the questionnaire used to collect data for the study. (DOCX 20 kb)

## Abbreviations

ANC: Antenatal care
aOR: Adjusted odds ratio
CI: Confidence interval
CSA: Central statistical agency
EDHS: Ethiopian Demographic and Health Survey
FIGO: International Federation of Gynecology and Obstetrics
HIV/AIDS: Human immunodeficiency virus/Acquired immunodeficiency syndrome
ICM: International Confederation of Midwives
MDGs: Millennium development goals
MOFED: Ministry of Finance and Economic Development
UN: United Nations
WHO: World Health Organization
