# Supplementary material for: Institutional delivery service utilization and associated factors among women of reproductive age in the mobile pastoral community of the Liban District in Guji Zone, Oromia, Southern Ethiopia: a cross sectional study
Source: BMC Pregnancy Childbirth. 2017 May 15;17:144. doi: 10.1186/s12884-017-1325-5 (PMC5433167; doi:10.1186/s12884-017-1325-5)
Supplement: Additional file 1: — Questionnaire. Title of data: English language questionnaire for study entitled “Institutional delivery service utilization and associated factors among women of reproductive age in the mobile pastoral community of the Liban District in Guji Zone, Oromia, southern Ethiopia: A cross sectional study”. Description of data: the questionnaire used to collect data for the study. (DOCX 20 kb) [file 12884_2017_1325_MOESM1_ESM.docx]

**English language questionnaire for study entitled “Institutional delivery service utilization and associated factors among women of reproductive age in the mobile pastoral community of the Liban District in Guji Zone, Oromia, southern Ethiopia: A cross sectional study”**

**Part I- socio-demographic characteristics**

| **S/N** | **Question** | **Possible response** | **Skip pattern** |
| --- | --- | --- | --- |
| 1 | How old are you? | ________(age year) |  |
| 2 | What is your current marital status? | 1. Single 2. Married 3.separated  4. Divorced 5. widowed | If not 2-go to 5 |
| 3 | What is your husband’s occupation? | 1.pastoralist  2.Farmer  3. other(specify)_____________ |  |
| 4 | What is your husband’s educational level? | __________(number of grade completed, put “0” if no attended class) |  |
| 5 | What is your occupation? | 1. House wife 2. Pastoralist  3. others(specify)___________ |  |
| 6 | What is your religion? | 1. Waqefata 2.Islam 3.protestant 4.Others(specify)_______ |  |
| 7 | What is your educational status? | 1. Literate (*confirm* )  2. Illiterate |  |
| 8 | Number of livestock owned by household ?(in number of “Hawicha”* ) | __________Cow  ___________camel  ________ __goat and sheep  ( write zero if null is owned) |  |
| 9 | How many months are elapsed since your household made last mobility? | ____ (convert all answer into equivalent month! if 5 years or more, put “ 5Y”) |  |
| 10 | How many person lives in the same household? | ___________ (number of person) |  |
| 11 | Do you usually listen to the radio? | 1. not at all 2. Some times  3 most of the times |  |

*" Hawicha"- is local livestock counting mechanism, in which only animals which gave at least one birth is counted.

**Part II: - obstetric history**

| **S/N** | **Question** | **Possible response** | **Skip pattern** |
| --- | --- | --- | --- |
| 21 | How many times, Have you ever given birth? | _____(number of all previous births) |  |
| 22 | Who makes decision to seek care for delivery service mostly | 1.Mainly wife  2.Wife and husband jointly  3.Mainly husband  4.Someone else |  |
| 23 | When you got pregnant of most recent birth, did you want to get pregnant at that time? | 1.yes  2.no |  |
| 24 | Did you see health professional for antenatal Care for the last pregnancy ended in most recent birth? | 1.yes  2.no |  |
| 25 | Where you gave birth of pregnancy preceding the most recent birth? | 1. Home 2. Traditional birth attendants home 3. Health post  4. Health center 5.Hospital  6.Others (specify) __________ | If no such birth go to part three |
| 26 | Was there any problem with that birth? | 1. Yes 2. no | If No go to part III |
| 27 | If yes to 26 what was the problem? | 1. Prolonged labor 2.stillbirth  3. Excessive vaginal Bleeding 4.convulsion 5.others(specify)_______ |  |

**Part III: - Institutional delivery use**

| **S/N** | **Question** | | **Possible response** | **Skip pattern** |
| --- | --- | --- | --- | --- |
| 31 | At a place when labor of most recent birth start, How many hours would it takes to reach the nearest health facility (i.e. Health post, health center, hospital, and clinic) from your home? | | ______hour/s and  ______ Minutes. |  |
| 32 | At a place when labor of most recent birth start, How many hours would it takes to reach the nearest functional transport road from your home? | | ______hour/s and  ______ Minutes. |  |
| 33 | At that time do you have any means of transportation to visit health facility? | | 1. Yes 2. no | If No go to 35 |
| 34 | If yes to 33, what was potential transportation available? | | 1. Human back 2. Animal back 3. Ambulance 4. Public transport 4. Other(specify)________ |  |
| 35 | When at the first time labor of most recent birth started, did you /your family have any ready available cash/money? | | 1.yes 2.no 3 .I do not remember  4.i do not know 5.other(specify)_____ |  |
| 36 | Where did you give birth of most recent birth? | 1. Home 2. Traditional birth attendant’s home 3.Health post 4. Health center 5.Hospital  6.Others (specify) __________ | |  |
| 37 | If answer to “36” is health institution, why you prefer there for delivery? | 1. I have preplanned to give birth there 2. I encountered problem during labor 3. Others(specify)_____________ | |  |
| 38 | Who assisted with the delivery of that birth? | 1.Nobody  2.Relatives  3.Traditional birth attendant  4.Health extension worker  5.Midwifes/doctor/nurse/health officer  6Other(specify)____________________ | |  |
| 39 | Was there any problem with that birth? | 1.no 2.yes | |  |
| 39.1 | If 39 is yes, what problem(more than one answer possible) | 1. Prolonged labor 2.stillbirth  3. Excessive vaginal Bleeding 4.convulsion 5.others(specify)_______ | |  |
| 39.2 | If answer for 36, is homes, why you did not deliver at health facilities? (multiple responses are possible) | 1.not necessary  2. not customary  3.I did not know existence of the service  4. Facility not open.  5 .too far/ no transportation  6. don't trust facility  7. no female provider at facility  8.husband/family did not allow  9. too costly  10. other (specify)________________- | |  |

**Part IV: - knowledge and attitude**

| 41 | Do you know that health institutions provide institutional delivery services? | 1.yes 2.no |  |
| --- | --- | --- | --- |
| 42 | Could institutional delivery services add positive value to mother’s health? | 1.Yes 2.no 3.I do not know |  |
| 43 | Do you believe that all pregnant women should give birth at health institution? | 1.Yes 2.no 3.I do not know |  |
| 44 | Do you believe that pregnancy by itself is risk condition | 1 .Yes 2.no 3. other(specify)_____________ |  |
| 45 | Do you want to deliver at health facility for your future birth? | 1 .Yes 2.no 3. other(specify)_____ |  |
| 46 | Do your families permit you, in case you decide to deliver at health institution? | 1.Yes 2.no 3.I do not know |  |
| 47 | Would you deliver at health facility where male provides services? | 1. Yes, no problem 2. yes ,if there is no option ,I will go 3.no ,I will not go 4. Other(specify)___________ |  |
| 48 | Do you know that ambulance is available at woreda office to take you to health facility if you are in labor free of charge? | 1. Yes 2. no |  |
| 49 | Do you know that government health institutions provide delivery services free of charge? | 1. Yes 2. no |  |
